# Balancing intellectual monopoly privileges and the need for essential medicines

**DOI:** 10.1186/1744-8603-3-4

**Published:** 2007-06-12

**Authors:** Greg Martin, Corinna Sorenson, Thomas Faunce

**Affiliations:** 1 Science and Research Department, World Cancer Research Fund, 19 Harley Street, London, W1G 9QJ, UK; 2 London School of Economics, LSE Health, Cowdray House, Houghton Street London, WC2A 2AE, UK; 3 College of Law, Medical School, Globalization and Health Project – Centre for Governance of Knowledge and Development, Australian National University, Australia

## Abstract

This issue of Globalization and Health presents a paper by Kerry and Lee that considers the TRIPS agreement and the recent policy debate regarding the protection of public health interest, particularly as they pertain to the Doha Declaration. In this editorial, we consider the debate, the conclusions thereof, and identify five questions that should be considered by key stakeholders in ongoing discussions.

## Background

The World Trade Organisation's (WTO's) agreement on Trade-Related Aspects of Intellectual Property Rights (TRIPS) has remained controversial ever since its inception at the behest of some of the world's largest multinational corporations. Balancing the need to protect the intellectual property rights (IPRs) (which the third author considers are more accurately described as intellectual monopoly privileges (IMPs)) of pharmaceutical companies, with the need to ensure access to essential medicines in developing countries is one of the most pressing challenges facing international policy makers today. In order for Commonwealth nations to craft and implement IPR (or IMP) legislation that realises this balance, decision-makers need to capitalise on the flexibilities and provisions afforded by the agreement, particularly compulsory licensing. Nonetheless, the industry-influenced US Trade Representative (USTR) routinely opposes the use of such flexibilities and, despite contrary injunctions in US law, has sought to restrict them in a series of bilateral putatively 'free' trade agreements.

Despite recent advancements in prevention and treatment in many regions of the world, diseases such as HIV/AIDS, tuberculosis (TB) and malaria continue to scourge the poorest and most vulnerable of the global population. The vast majority of those suffering from these diseases live in developing countries, where low wages, high pharmaceutical prices and poor access to medical services means there is limited, if any, access to many of the life-saving drugs currently available in industrialised countries. In fact, about one-third of the world's population does not have access to essential medicines. Currently, 80 percent of the world's population lives in developing countries, but consumes less than 20 percent of all pharmaceuticals.

The problem of access to essential medications for the developing world is two-fold. First, research and development (R&D) is principally being driven by market forces, not medical need, when considered in light of estimates of the global burden of disease. Specifically, problems typically inherent to the industrialised world (e.g. impotence, obesity and baldness) are being prioritized over diseases that disproportionately affect the poor, such as TB and malaria. Indeed, 90 percent of the burden for global disease is carried by a population for whom only three percent of the R&D expenditure is directed. Of the 1,223 new chemical entities developed between 1975 and 1996, only 11 were for the treatment of tropical diseases. Increasingly, many large pharmaceutical corporations are not even doing much of in-house R&D, but simply doing venture capital searches for small biotechs to acquire.

Second, high prices for brand name and patented pharmaceuticals often create a barrier to access in developing countries. Patent monopoly protection of new drugs allows the inventing company sufficient time to recoup their controversially-estimated R&D costs. Sponsors, however, often seek extra patent reward for innovation via a number of existing 'loopholes'. For example, companies often use bilateral trade agreements to eliminate reference pricing that bases the price of a new drug on pharmacoeconomic evidence, such as its efficacy, safety, and cost-effectiveness relative to comparable existing therapies. Such tactics make patented medications prohibitively expensive for people living in poorer countries. As a result, international trade agreements have become an exceedingly important issue for access to essential medicines and health services.

Several multilateral agreements established by the World Trade Organization (WTO), the central body governing international trade, impact public health. Of these agreements, 'Trade-Related Aspects of Intellectual Property Rights' (TRIPS) most significantly influences trade policy in the pharmaceutical sector and global access to essential medicines. One broader concern of the TRIPS agreement is that the World Health Organisation (WHO) has only non-voting observer status on the principal WTO policy organ and most key WTO documents make no reference to international economic, social and cultural rights, such as the right to health in article 12 of the ICESCR (International Covenant on Economic, Social and Cultural Rights).

## Background on the TRIPS agreement

The TRIPS agreement, negotiated at the end of the Uruguay Round of the 'General Agreement on Tariffs and Trade' in 1994, sets forth global minimum standards for protecting and enforcing all forms of IPRs (or IMPs), including those for pharmaceuticals. The agreement contains a number of requirements that WTO member countries must satisfy in their national laws. While all countries are required to incorporate TRIPS standards, provisions were stipulated to allow developing countries until 2006 and least-developed countries until 2016 to implement these changes. While the agreement's 73 articles cover a broad range of topics, there are several key requirements related specifically to pharmaceuticals. In return for agreeing to these standards (given they had little endogenous pharmaceutical manufacturing capacity), developing nations were informally promised greater access to developed world agricultural markets. This has never eventuated, so the failure to make TRIPS accession by such developing nations formally conditional on such *quid pro quo *appears to have been a gross negotiating error.

### Exclusive patent protection and minimum 20-year term

A drug that is patented can only be made, used, imported/exported or sold by the patent holder, subject to certain public interest exceptions. Under the TRIPS agreement, governments are required to recognise patents on products and processes in most areas of technology and to confer rights to the patent holder for a given period of time. This stipulation serves to protect the patent holder from another party making, selling or importing the drug during the period it is still under patent. Frequently, patent protection results in significantly higher prices for patented medicines than in the context of market competition.

### Rights conferred

While TRIPS upholds the rights granted to a patent holder, it allows for limited exceptions and provisions, including compulsory licensing, protection of proprietary information, protection of trademarks and enforcement. This recognises that patents are not a natural form of human rights, like freedom of expression or association, but are really a monopoly privilege granted by society in return for speedy access to and dissemination of important knowledge. Indeed, it is this public interest benefit from patents that has been so little supported by an agreement such as TRIPS, particularly in the area of compulsory licensing.

### Non-discrimination

The TRIPS agreement requires that countries make patents and all patent rights available 'without discrimination' on certain grounds. Under TRIPS, countries are not allowed to treat national and foreign inventions differently, nor can their respective patent laws discriminate between imports and domestic products. Moreover, pharmaceutical patent holders cannot abuse their dominant position in contractual relationships to prohibit competition. TRIPS also prohibits countries from creating legislation that discriminates against any area of technology. Subsequent WTO trade panel decisions have clarified that this latter prohibition does not apply where problems arise regarding a particular area of technology, as demonstrated by the Canadian Generic Medicines case on patent 'evergreening'.

## TRIPS and public health

The TRIPS agreement indicates that patent rights need to be balanced against other important interests, such as public health. In shaping their laws to conform to TRIPS standards, countries 'may take measures necessary to protect public health'. Standards are not always appropriate for poor countries with a multitude of health and development demands, developing countries can employ certain TRIPS provisions to protect public health. Such options relevant to accessing essential medicines include: exclusions to patentability; exceptions to patent rights; parallel importing; compulsory licensing and promotion of generic drugs.

Despite these provisions, concerns existed surrounding the impact of TRIPS on access to essential medicines, particularly in developing countries. These issues were addressed in June 2001 at the WTO Ministerial Conference in Doha, Qatar, when WTO ministers confirmed that the TRIPS agreement should be implemented and interpreted in a way that supports public health and promotes access to medicines. This agreement was captured in The *Doha Declaration on TRIPS and Public Health*, which affirmed the sovereign right of countries to take measures, particularly through compulsory licensing and parallel imports, to protect public health and give it priority over intellectual property. Specifically, the declaration states that the TRIPS agreement 'does not and should not prevent members from taking measures to protect public health... (and it) should be interpreted and implemented in a manner supportive of WTO members' right to protect public health and in particular, to promote access to medicines for all'. Of particular interest was the interpretation of Article 31(f) of the TRIPS agreement, which states that compulsory licensing shall be 'predominantly for the supply of domestic markets'. To this end, governments can issue a license to a local manufacturer, whilst offering a lower level of compensation to the originator. In effect, compulsory licensing essentially lowers prices to consumers by creating competition in the market for the patented product. As many developing countries lack the domestic capacity or technical expertise to manufacture patented pharmaceuticals, the WTO further agreed to modify the TRIPS provisions relating to compulsory licensing. In August 2003, a temporary waver was established to permit countries with local manufacturing the ability to issue compulsory licenses and export drugs to countries unable to produce pharmaceuticals domestically. In December 2005, the provision was confirmed as an amendment to the TRIP agreement. Until such changes go into effect in December 2007, the temporary waiver will apply.

The aforementioned actions demonstrate that some of the issues surrounding the high costs of and limited access to medicines in developing countries can be offset by regulatory measures. However, the existence of such provisions is not sufficient in and of itself. Effective protection of public health also depends upon successful implementation, as discussed below.

## Implementation of the TRIPS agreement provisions

Despite the flexibilities granted by the TRIPS agreement, few developing countries have employed them to develop policies to protect public health. Consequently, organisations such as Medecins Sans Frontieres and Oxfam argue that the provisions have had minimal impact on improving access to affordable medicines for the world's poor. Effective implementation of such provisions, most notably compulsory licensing, has been adversely affected by several factors.

First, the implementation of compulsory licensing is complex and requires a sufficient local administrative infrastructure, which is often prohibitively expensive, with costs in excess of US$1.5 million. Second, the flexibilities provided by the TRIPS agreement have been limited by a number of recent developments, such as bilateral and regional free trade agreements. Free trade agreements, negotiated by the USTR industrialised countries (e.g., Australia and South Korea), require commitments beyond those specified by TRIPS. Such provisions include 1) extending patent terms beyond 20 years for delayed marketing approval, 2) limiting parallel imports of patented drugs, restricting grounds for compulsory licensing, 3) imposing 'data exclusivity' rules, 4) defining 'innovation' to include minor 'me-too' molecular variations, 5) facilitating elimination of reference pricing, and 6) introducing the pro-evergreening 'linkage' of safety and quality regulatory assessments of proposed new generic market entrants with patent checks. The new patent laws in India highlight the implementation of such 'TRIPS-plus' provisions. Some commentators, such as the third author, however, using criteria more closely related to public health and relieving the global burden of disease, prefer to call such arrangements with their valorizing of IMPs, 'TRIPS-minus'.

Despite the challenges, several countries have taken steps to use compulsory licensing and other flexibilities, such as government-use orders. Examples of these steps include:

• Malaysia issued a government-use order and granted a contract to a local firm to import three HIV/AIDS drugs from an Indian manufacturer for supply to government hospitals;

• The Zimbabwe government declared a health emergency with regards to HIV/AIDS and, subsequently, issued compulsory licenses to three local companies to either import or produce antiretroviral drugs; and,

• Canada recently passed legislation to allow Canadian manufacturers to export antiretroviral drugs to countries lacking production capacity. Royalties paid to the patent holder vary according to the importing country's Human Development Index. Despite strong good will from Canada's largest generic drug manufacturer (Apotex), however, it has proved extremely difficult to develop a product that would satisfy the exception.

• Having failed in prolonged negotiations with patented drug manufacturers to obtain prices low enough to allow them to provide essential medicines to meet significant public health needs, Thailand and Brazil have recently issued compulsory licenses over HIV/AIDS medications in the face of strong opposition from the US Trade representative.

All of these measures have been actively opposed by pharmaceutical multinationals and the USTR, with the WHO less than forthright in extending strong support.

## Utilising TRIPS: the issues and actions involved

To ensure that governments and policy-makers effectively utilise the alternatives offered by the TRIPS agreement, there are several issues and actions to consider. First, the TRIPS agreement allows countries to use either strict or flexible criteria for patentability. If flexible criteria for novelty and inventiveness are implemented, pharmaceutical companies may apply for patents for different formulations of known drugs and thus expand the duration of the protection beyond that of the original patent. Therefore, it is important that health ministries work together to formulate and/or revise their national patent legislation to promote public health goals. TRIPS provisions can also be used to stimulate access to generic medications, depending on the way in which national legislation is designed. For example, countries may allow for testing and regulatory approval of generic versions of drugs before the patent has expired, thereby allowing availability of generics soon after patent expiration. Additionally, national patent legislation can be crafted to incorporate exceptions, trademark provisions and other measures to support generic competition.

In order to facilitate the prompt introduction ('spring boarding') of generic medicine following the expiration of patents, governments may enact 'Bolar exceptions', whereby competing companies can make applications for the development and approval of a generic product before the patent expiration date. In November 1998, the European Commission and their member states requested that the WTO Dispute Settlement Body establish a panel to examine the application of the Bolar provision in the 'Canadian Patent Act'. The panel concluded that Canada was in violation of their obligation under TRIPS by stockpiling pharmaceuticals during the six months prior to the expiry of the patents, but they were not in violation in allowing the 'spring boarding' development and submission of information required to obtain marketing approval for products without the consent of the patent holder.

As previously discussed, the TRIPS agreement does not limit the grounds or reasons for issuing a compulsory license. Nonetheless, USTR-negotiated bilateral free trade agreements, such as those with South Korea and Australia, attempt to limit it to 'national emergencies of extreme urgency.' Compulsory licensing should be allowed to be employed to the full (in accordance with the *Doha Declaration on TRIPS and Public Health*) to encourage access to medications. However, the ways in which compulsory licensing can be employed should be clearly defined and, to reduce the risk of abuse, should be used only in accordance with existing laws. Moreover, issues related to sufficient patent protection and remuneration should be considered to encourage the increased use of compulsory licensing and maximize competition. Broadly, the WHO recommendation that developing countries exercise caution in enacting legislation that is more stringent that the TRIPS agreement requirements should be strengthened. Countries pressured into making such concessions during bilateral trade negotiations should be supported in interpreting their commitments in the light of the then extant *Doha Declaration on TRIPS and Public Health*. An encouraging sign in this regard is that the US Congress, as a condition of renewing 'fast-track' (yes or no) approval of trade agreements, required the USTR to stop pushing for limitations to compulsory licensing, 'linkage' evergreening, and patent term extensions for delayed marketing approval. This leaves the door open for renegotiation of those prior bilateral trade agreements with the US that have included provisions limiting access to essential medicines.

## Monitoring the TRIPS agreement

The TRIPS agreement has proven to be one of the most controversial WTO agreements and the public health impacts are still relatively unclear. As such, continued monitoring of the agreement is necessary to analyse its effects. Key questions to be raised include:

1. Should the TRIPS agreement be renegotiated to allow new drugs for historically neglected diseases to be more rapidly and effectively developed?

2. Is the introduction of ultra-low cost generic medicines the answer or does this only ride on the lower environmental standards of factories in China and India, which source the world's active product ingredients (APIs)?

3. Are the costs of essential drugs becoming affordable or prohibitively expensive on a global scale? What regulatory changes could be encouraged to ensure that access to both quality generic and new patented medicines does not become a two-tier problem with different drivers for rich and poor?

4. Are transfers of technology and direct foreign investment in developing countries increasing or decreasing? What impact is this having on the burden of disease?

5. Are poor countries able to implement the provisions within the TRIPS agreement to protect public health? What changes can be made to TRIPS to facilitate effective implementation?

As evidenced, the role of IPR (or IMP) trade policy on public health is of increasing relevance. Understanding the TRIPS agreement is important for creating the legal tools and public policies necessary to capitalise on the potential offered by its provisions. In this context, it is crucial to maintain the flexibilities established by the agreement, especially with regards to developing countries, in order to achieve greater equity in accessing medicines. To reach this end, strong relationships between health ministers and other governmental bodies should be established to develop a unified strategy for effective policy development.

